# Improving Data Aggregation in IoT Sensor Networks Using Self-Organizing Maps and Firefly Optimization Algorithm

**DOI:** 10.3390/s25237107

**Published:** 2025-11-21

**Authors:** Hassan Sh. Alshehri, Fuad Bajaber

**Affiliations:** Department of Information Technology, Faculty of Computing and Information Technology, King Abdulaziz University, Jeddah 21589, Saudi Arabia; fbajaber@kau.edu.sa

**Keywords:** data aggregation, Internet of Things, sensor networks, firefly algorithm, SOM, optimization

## Abstract

Internet of Things (IoT) sensor networks comprise diminutive sensor units primarily designed for monitoring phenomena within a designated area. However, reaching the complete potential of this kind of network is extremely difficult due to several challenges, including the fact that the data transmitted by the sensor nodes contains a large amount of duplicates. Data aggregation can be employed to address this issue in routing packets from nodes that send data to the base station (BS). In this study, a novel, hybrid data aggregation framework for IoT sensor networks is proposed by integrating Self-Organizing Maps (SOMs) with the Firefly Optimization Algorithm (FOA). The core motivation for this integration is to address persistent challenges in IoT sensor networks, chiefly energy efficiency, network longevity, and the reliability of data transmission. By combining the adaptive, unsupervised clustering capabilities of SOMs with the robust, multi-objective optimization properties of the FOA, the method aims to achieve more intelligent, adaptive, and practical solutions for real-world IoT systems. This work presents an innovative framework that synergistically leverages the strengths of FOA and SOM, offering a new methodology that addresses key challenges in scalable and energy-efficient IoT sensor network clustering. The suggested algorithm’s validity has been verified using an experimental analysis performed in MATLAB. Experimental results show the proposed method extends network lifetime by 15% and reduces energy consumption by 10% compared to FOA, SOM, and LEACH benchmarks. A notable classification rate was attained after implementing and testing the proposed method using the Intel Berkeley Research Lab dataset.

## 1. Introduction

The rapid expansion of IoT deployments has fundamentally changed how data is gathered, processed, and sent across distributed sensor networks. Contemporary IoT applications, including smart cities and industrial monitoring systems, produce extensive volumes of diverse data, necessitating effective aggregation methods to guarantee optimal network performance, energy efficiency, and data integrity. Data aggregation has become an essential process that entails systematic collection, processing, integration, and transmission of data from various distributed sensor nodes to diminish redundancy, reduce energy consumption, and improve the overall network performance while preserving data integrity and quality of service standards.

The importance of efficient data aggregation in IoT sensor networks is particularly clear when acknowledging the inherent constraints of conventional Wireless Sensor Networks (WSNs), such as limited battery life, restricted computational resources, bandwidth constraints, and the necessity to sustain network connectivity despite node failures and environmental fluctuations. Recent studies indicate that cluster-based data aggregation methods can markedly improve the network efficiency and lifetime. Notably, ref. [1] introduced a cluster-based data aggregation framework tailored for IoT sensor networks by employing the FOA. This approach demonstrated significant enhancements in energy efficiency and network performance relative to traditional clustering techniques. This study laid the groundwork for bio-inspired optimization methods in IoT data aggregation by illustrating how the natural luminescent activity of fireflies may be converted into efficient clustering and routing strategies.

Expanding the use of firefly optimization to tree-based energy-efficient data aggregation techniques in IoT settings introduced a hierarchical strategy that enhances both data-routing pathways and energy consumption patterns. The tree-based approach [2] tackles essential issues with data transmission latency and energy distribution across network nodes, showcasing notable enhancements in network longevity and data delivery efficacy. This study emphasized the adaptability of firefly optimization in managing various network topologies and aggregation procedures, facilitating the development of more advanced optimization methods in IoT sensor networks. The researchers used adaptive brightness parameters that are altered dynamically according to node energy levels and data traffic patterns, leading to more equitable energy consumption throughout the network hierarchy [2].

The substantial validation of firefly-based optimization in WSNs has been demonstrated through various research initiatives. Mosavvar and Ghaffari [3] performed an extensive study on data aggregation with the Firefly Algorithm (FA) in conventional WSNs, defining foundational principles and performance metrics that have impacted later research in IoT applications. In the suggested methodology, sensor nodes are separated into multiple regions by clustering. Within each cluster, nodes exhibit periodic activity and inactivity. Factors such as energy and distance are considered in the selection of active nodes. Consequently, nodes with greater residual energy and increased distance will be identified as active nodes [3].

In addition to the optimization capabilities of the FOA, SOMs have emerged as potent unsupervised learning instruments for data grouping and pattern detection in sensor networks [4,5]. SOMs present distinct benefits in data aggregation contexts by employing adaptive clustering techniques that proficiently categorize analogous data patterns while minimizing the dimensionality and computing demands [6,7]. The use of SOMs in WSNs has demonstrated favorable outcomes across multiple applications, such as energy-efficient clustering algorithms [7,8], distributed data processing [9], and network security enhancement [10].

Although FOA and SOM strategies have individually succeeded in optimizing various facets of sensor networks, a substantial research gap remains in utilizing their combined capabilities for enhancing data aggregation comprehensively. The integration of these complementary approaches presents an opportunity to create hybrid solutions that can simultaneously optimize multiple network parameters while maintaining adaptive learning capabilities [11]. This combination may overcome the shortcomings of current methods by merging the global optimization advantages of FOA with the pattern recognition and clustering abilities of SOMs.

The examination of the FOA indicates that it is incapable of identifying optimal solutions. Furthermore, when firefly particles become ensnared in the local minimum, they are unable to get rid of it and exhibit a diminished convergence rate. To address these limitations, we have suggested the integration of a SOM and the FOA. Consequently, the rate of convergence and the precision of the outcomes have improved; also, the issue with the convergence of solutions to local optima and the entrapment in local minima has been largely addressed. This research aims to explore the beneficial potential of integrating SOMs with the FOA to develop an improved data aggregation framework for IoT sensor networks and overcome the significant issues of energy efficiency, network longevity, and data transmission reliability in contemporary IoT implementations by taking advantage of the multi-objective optimization capability of FOA and the adaptive clustering capability of SOMs. The proposed method is anticipated to enhance intelligent data aggregation approaches and offer realistic solutions for real-world IoT applications.

The subsequent sections are structured as follows: Section 2 analyzes relevant literature. The proposed method is detailed in Section 3. Section 4 establishes the results and discussion derived from simulating the suggested model and contrasts this model with alternative models. Ultimately, Section 5 delineates the conclusion of the research.

## 2. Related Works

The topic of efficient data aggregation in IoT sensor networks has garnered significant research interest because of its essential function in enhancing network performance, prolonging the network lifespan, and minimizing energy usage. The clustering paradigm has arisen as a crucial method for data aggregation in sensor networks. The integration of multiple optimization strategies has demonstrated encouraging outcomes in tackling intricate data aggregation issues. Mohammadi et al. [11] introduced FA-SOM, a hybrid methodology that employs self-organizing neural networks as clustering techniques, augmented by optimization through the FA. The firefly optimization process initially identifies cluster centers using data-clustering operations, subsequently adjusting the initial weights of the SOM neural network based on the identified cluster centers [11].

SOMs have arisen as a potent unsupervised machine learning method for data aggregation in WSNs. Abdalkafor et al. [5] performed an extensive study on the implementation of SOM, Hierarchical Agglomerative Clustering (HAC), and Radial Basis Function (RBF) algorithms for data aggregation in WSNs. Their research revealed that an SOM offers enhanced clustering efficacy when integrated with suitable preprocessing techniques, such as balanced distribution methods and normalization processes [5]. The efficacy of SOMs in managing high-dimensional complex datasets has been especially significant in WSN applications. Ihsan et al. [4] introduced an innovative data aggregation approach utilizing SOMs that was aimed at minimizing redundant data and eradicating outliers in WSN environments. Their methodology utilized cosine similarity metrics to enhance the grouping of sensor nodes according to the data density and similarity attributes. The application of interquartile range (IQR) analysis for outlier elimination was crucial in improving the data quality prior to the SOM-processing phase [4].

The FA has proven to be exceptionally effective for addressing NP-hard optimization issues associated with WSN clustering and data aggregation. Zivkovic et al. [12] devised an updated iteration of the FA aimed at optimizing the longevity of WSNs by refining cluster formation and Cluster Head (CH) selection methodologies. Their research indicated that metaheuristic algorithms, especially the enhanced FA, exhibit superior and more consistent performance relative to conventional methods, such as LEACH, the basic FA, and particle swarm optimization, when assessed on identical network infrastructure models. Pakdel and Fotohi [13] presented the EM-FIREFLY approach, employing the FA for power management in WSNs. Their methodology tackled the significant problem of non-homogeneous power consumption, especially impacting nodes adjacent to base stations that undergo accelerated energy depletion due to continuous data transmission activities. The method utilized four principal criteria: residual energy, noise rate, number of hops, and distance to identify optimal CHs with high desirability based on fitness functions [13].

The integration of multiple optimization algorithms has shown potential for addressing complex routing challenges in IoT networks. Hosseinzadeh et al. [14] introduced a hybrid delay-aware clustered routing methodology that integrates the Aquila Optimizer and FA to tackle the NP-hard characteristics of clustered routing issues. Their research revealed that conventional cluster-based routing techniques frequently experience disproportionate power consumption, resulting in early network failure. The authors devised a clustering strategy based on swarm intelligence that attained a more equitable distribution of cluster heads, leading to enhanced energy efficiency. The study emphasized the necessity of accounting for many aspects, such as residual energy, average distances, hop count, and node equilibrium, in the design of routing protocols for IoT platforms [14].

Abdalkafor and Aliesawi [15] performed an extensive investigation illustrating the substantial effects of data aggregation algorithms on the performance of WSNs regarding energy efficiency, accuracy, and latency. Their study employed SOMs and HAC algorithms with the Intel Berkeley Research Lab Dataset, thereby creating baseline performance measures for assessing aggregation efficacy [15]. The integration of fuzzy logic in WSN data aggregation has demonstrated encouraging outcomes. Bhushan et al. [16] devised the Fuzzy Attribute-based Joint Integrated Scheduling and Tree Formation (FAJIT) method, which resolves parent node selection issues in heterogeneous networks. Their methodology employs fuzzy logic for tree construction and parent node selection, leveraging min–max normalization to provide normalized weights for graph edges, hence enhancing the energy efficiency relative to conventional distributed techniques [16]. The quality and efficiency of the incoming data are improved by obtaining aggregated data that is free from errors and noise. This necessitates the application of precise algorithms that efficiently eliminate mistakes and noise from the data before aggregation, such as the Accurate Data Aggregation Created by Neural Network and Data Classification Processed Through Machine Learning (ACNM) technique [17] for WSNs, which aims to improve data accuracy and efficiency by eliminating errors and noise before aggregation. It reduces the network delay and overhead by optimizing computation periods, reducing redundant data transmissions, and controlling the transmission rate [17].

Recent advancements have unveiled more advanced clustering methodologies that tackle deployment structure issues. Krishnasamy et al. [18] introduced a heuristic angular clustering framework for secure statistical data aggregation, offering Radial-Shaped Clustering (RSC) as an alternative to conventional square or rectangular deployment configurations. The RSC methodology categorizes deployment architecture into several virtual concentric rings, each of which is further partitioned into sectors that operate as clusters. The node nearest the middle of each sector is designated as the CH, and data aggregation is executed using angular inclination routing [18].

Researchers have established weighted K-means clustering as an improved methodology. Surya and Suresh [19] introduced an extended weighted K-means algorithm integrated with key performance indicators for data aggregation challenges. Their methodology acknowledges that each variable in a dataset possesses varying degrees of significance, and clustering results can be markedly enhanced by allocating suitable weights that comprehensively reflect their impact on cluster dynamics. This methodology employs a feature evaluation function to produce vectors derived from feature weights via reduction functions, framing the problem as a multi-objective optimization challenge [19].

Several clustering-based strategies have been devised to enhance data aggregation efficiency. Tanushree et al. [20] introduced a hierarchical cluster-based data aggregation method using fault tolerance features to enhance network longevity. Their methodology employs LEACH clustering with numerous drains to mitigate communication costs and avert network isolation after CH failures. The strategy employs Virtual Cluster Heads (VCHs) as auxiliary nodes to provide uninterrupted data transfer in the event of primary cluster head failure [20]. Energy-efficient clustering systems have been augmented by diverse optimization techniques. Roy et al. [21] concentrated on minimizing intra-cluster communication by permitting non-cluster head nodes to send control frames prior to comprehensive data delivery to designate cluster heads, thereby exhibiting enhanced stability and network longevity in LEACH simulations [21]. Al-Humidi et al. [22] created a lightweight data transmission (LDT) system employing centralized control algorithms for optimal cluster head selection, functioning in rounds with specific phases for initialization, clustering, aggregation, and data transmission [22].

Advanced routing and aggregation techniques have integrated multi-objective optimization strategies. Sharifi and Barati [23] introduced a two-level energy-efficient routing and clustering approach that incorporates many factors, such as residual energy, centrality, and proximity to the sink for the selection of CHs. Their methodology establishes rendezvous zones with backbone nodes that create tree structures for effective data transmission, providing two separate methods for data transmission contingent upon the location of the source CH in relation to the sink [23]. Particle Swarm Optimization (PSO) has been utilized for clustering and routing challenges in sensor networks. Ruan and Huang [24] formulated a PSO-based Uneven Dynamic Clustering Multi-hop Routing Protocol (PUDCRP), which utilizes enhanced PSO algorithms to dynamically ascertain CH positions and facilitates energy-efficient routing via multi-faceted fitness functions [24]. Wang et al. [25] introduced Energy Centers Searching using PSO (EC-PSO) to mitigate energy depletion and identify appropriate energy centers for CH selection, employing varied techniques throughout network phases.

Moreover, Manuel et al. [26] emphasized that current sensor network research incorporates a variety of technologies to enhance previous studies that focused on cost-effectiveness, time efficiency, and new approaches. The authors recognized deficiencies in prior models, especially regarding the necessity for improvements in energy management inside WSNs. The study classified its technique into three primary categories: optimization-based, parameter-based, and methodology-based. The authors made it a priority to integrate the energy limitations of sensor hubs and the clustering model features as an effective strategy for maintaining the power efficiency of WSNs [26]. Table 1 represents a summary of the related works.

Study [27] employed a meta-heuristic known as the FA and contrasted its outcomes with those of the established Energy Aware clustering algorithm. The application of the firefly method in clustering shows enhancements in performance metrics, such as the packet delivery ratio between network nodes and the network longevity. The improvement in the packet delivery ratio, relative to the basic approach, signifies a decrease in packet loss within the network. A similar study [3] indicated that the lifespan of WSNs can be extended through the selection of appropriate CHs and the application of the FA as a data aggregation method in WSNs. The proposed strategy entailed segmenting sensor nodes into distinct regions by applying clustering algorithms. Each cluster exhibited a pattern of alternating inactive and active nodes, with the selection of active nodes determined by factors such as energy and distance. The methodology utilized in this study involved identifying nodes that demonstrated increased distance and elevated residual energy as active nodes.

Study [12] presented an improved variant of the FA to extend the network’s longevity. This version enhanced the processes of cluster formation and CH selection. Furthermore, they performed an evaluation to determine the efficacy of the improved FA, contrasting LEACH, the foundational FA, with particle swarm optimization. All algorithms were assessed using the same network architectural framework, and simulations demonstrated that the proposed metaheuristic surpasses rival algorithms in both performance and consistency. Study [13] employed the FA and assessed its efficacy according to four criteria: noise level, residual energy, distance, and hop count. The EM-FIREFLY approach delineates the selection of the most optimal CH by utilizing a fitness function and transmits data packets through these CHs to the sink. The evaluation outcomes indicate the effectiveness of the EM-FIREFLY approach concerning the criterion of maximum comparative load and network lifespan.

In another study, researchers proposed a firefly optimization method and an aquila optimization methodology to identify the requisite interim and CH nodes for transportation, acknowledging the NP-hard nature of clustered route construction. Researchers have evaluated the efficacy of this hybrid clustering and transmission methodology with the following metrics: residual energy, average distances, hop count, and node balancing. The proposed method has demonstrated efficacy, yielding at least an 18% enhancement in the system energy consumption and a minimum 25% augmentation in the packet delivery ratio [14]. The authors of [28] utilized the FA and hesitant fuzzy approaches to propose a unique CH selection strategy. The suggested method employs three qualities of sensor nodes to calculate the score of each individual node for the purpose of identifying the ideal CHs. Three instances were developed and evaluated to characterize the performance of the proposed strategy. The simulation results demonstrate that the proposed strategy improves the energy efficiency and prolongs the network lifespan.

In another investigation, the researcher presented an innovative data aggregation technique by employing the FOA to reduce the energy usage and extend the network’s lifespan. This study explores the correlation between the BS, which acts as the tree’s root, and the mediator node, which operates as a collector of its subordinate nodes. The goal is to construct an aggregate tree that reduces the distances between nodes. This technique yielded superior outcomes for the energy consumption, network durability, packet delivery ratio, and latency when compared with the Harris Hawks optimization and R-Leach algorithms [2]. A viable strategy to mitigate the challenges associated with energy consumption and network longevity entails the judicious selection of CHs within each cluster. The open shortest path routing protocol is employed to identify the route or link that expends the minimal energy or load necessary for data transmission between nodes. This study presents a hybrid method that integrates particle swarm optimization (PSO) with the FA for energy optimization. This hybrid technology conserves network power, hence prolonging the network lifespan [29].

Although widely applied, the Firefly Algorithm has faced substantial criticism. Scholars argue that the FA and other metaphor-driven heuristics, such as the Grey Wolf Optimizer and Bat Algorithm, rely on reformulations of previously established principles, especially those underlying PSO [30,31]. Critics further contend that such algorithms often introduce redundant terminology without offering robust theoretical justification for their design [32].

Nevertheless, contemporary hybrid metaheuristic research suggests that the FA’s real value lies less in its standalone application and more in its capacity to enhance other algorithms through integration. By merging distinct optimization methods, hybrid systems leverage the complementary strengths of each. For example, coupling the FA’s dynamic exploration–exploitation mechanisms with the structural learning capabilities of SOMs enables the maintenance of topological data integrity while adaptively optimizing clustering parameters for both global search diversity and local refinement.

In this study, the FA is not presented as a novel or independent solution but rather as a practical heuristic within a hybrid FA–SOM framework designed for adaptive and energy-efficient data aggregation. The integration takes advantage of the FA’s simplicity and adaptability alongside SOM’s stable spatial clustering, overcoming each method’s limitations when used in isolation. This hybrid approach is particularly suitable for dynamic environments, such as IoT sensor networks, where network and data conditions frequently change. The resulting model offers self-regulation, topology-aware optimization, delivering reliable aggregation performance in complex, data-driven contexts.

WSNs apply various data aggregation techniques aligned with specific network characteristics and requirements. Each protocol offers distinct advantages and limitations. Table 2 provides a comparative overview of existing data aggregation strategies alongside the proposed hybrid SOM-FOA methodology, demonstrating its relative strengths in energy efficiency and accuracy.

## 3. Proposed Method

This paper proposes an innovative hybrid framework that integrates SOMs and FOA to tackle the critical issues of energy efficiency, network lifetime, and data transmission reliability in IoT sensor networks. The framework leverages the adaptive clustering features of SOMs and the multi-parameter optimization capabilities of FOA to efficiently aggregate and transmit sensor data, with the objectives of reducing redundancy, optimizing resource use, and improving real-world applicability in contemporary IoT environments. In the initial phase of the proposed methodology, SOMs are employed to dynamically cluster sensor nodes according to their spatial locations, commonalities in sensed data, and current resource conditions, including energy levels and connectivity. In contrast to static clustering methods, SOMs dynamically restructure the network into clusters that correspond to variations in the node distribution, data patterns, and network conditions. This yields clusters that are optimally balanced regarding the load and spatial distribution, which is essential for reducing duplicated transmissions and maximizing the network resource efficiency.

Following the formation of clusters using the SOM method, the next phase utilizes the FOA to pinpoint the best CHs within each cluster. The FOA is ideally equipped for this purpose owing to its capacity to identify optimal solutions inside intricate, multi-parameter environments. The FOA assesses each prospective node through a fitness function that integrates essential criteria, including the residual energy, signal-to-noise ratio, distance to the base station, and the number of hops needed for data transmission. The FOA’s light attraction mechanism is adapted so that nodes with higher fitness—those more suited to serve as CHs—attract other nodes and are more likely to be selected. This dynamic and distributed optimization ensures that CHs are chosen not just for their location but for their ability to prolong the network lifetime and maintain robust communication links. CHs are responsible for aggregating data from their respective cluster members. The CHs subsequently transmit the consolidated data to the BS.

### 3.1. Firefly Optimization Algorithm

**Figure 1:**
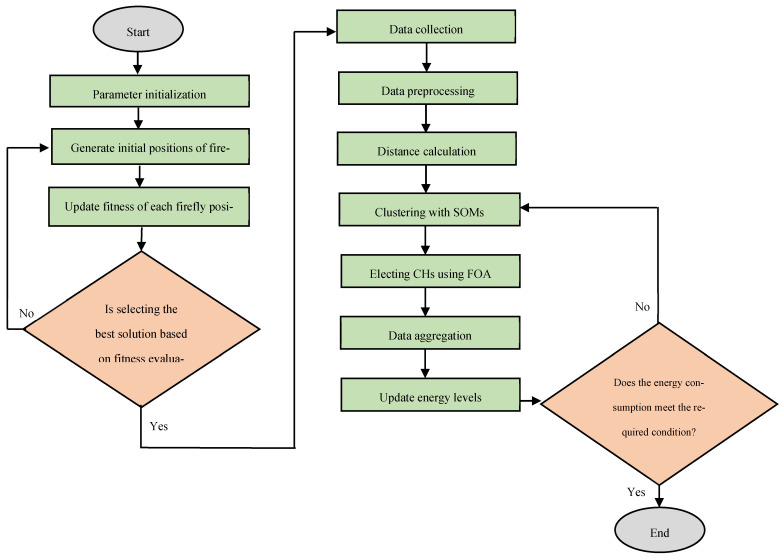
Flowchart of the suggested approach.

The suggested technique integrates the flexible grouping capabilities of SOMs with the optimization powers of the FOA to efficiently collect and transmit sensor data, with the objective of reducing superfluous data, optimizing resource utilization, and improving practical applications in contemporary IoT environments. By using sensor nodes analogous to fireflies and light intensity (fitness) as a measure of solution quality, the FA is employed to identify a specific number of nodes that have at least one neighboring node within a threshold distance, therefore facilitating the strategic election of CHs. The array (lightIntensity) stores the fitness (light intensity) of each firefly. The light intensity (fitness) of each firefly is determined by evaluating the intended function. An exceptionally luminous firefly interacts with others inside the specified search space to identify the optimal solution. Figure 1 illustrates the flowchart of the proposed methodology. The fitness value is a variable parameter derived from the residual energy, signal-to-noise ratio, inter-node distance, and *HoP* count. The algorithm progressively adjusts the locations of the fireflies to enhance the quality of the solutions (Equation (1)). The election of the CHs is executed in a completely decentralized manner using a fitness function. This technique employs a principle of great attraction to identify an appropriate CH. Prior to calculating the fitness function, it is necessary to determine the residual energy, signal-to-noise ratio, inter-node distance, and *HoP* count (Equations (2)–(5)). Consequently, the objective fitness function for nodes demonstrating the highest attraction is calculated in accordance with Equation (6).

(1) $$X_{i}=x_{i}+\beta_{0}e^{\gamma r^{2}}\;\left(x_{j}-x_{i}\right)+a\left(rand-\frac{1}{2}\;\right)$$

Each solution $X_{i}$ is verified against every solution $x_{j}$ , j = 1, 2, …, i , starting from j = 1. $\beta_{0}$ = 1 which represents the attractiveness, and *γ* = 1 which illustrates the absorption coefficient for all simulations. a is a parameter utilized for randomization, where a ∈ [0, 1]. In our methodology, the value of a is 0.2. *r* represents the distance between firefly *i* and firefly j .

(2) $$E=\left[\frac{R_{e,i}}{\sum_{i=1}^{N}R_{e}}\right]$$

which gives the residual energy of node *i* with relation to the aggregate residual energy of all nodes within the cluster. $R_{e,i}$ represents residual energy of node *i*. *N* illustrates total number of nodes in the cluster.

(3) $$S=\left[\frac{SINR_{i}}{\sum_{i=1}^{N}SINR_{i}}\right]$$

which gives the signal-to-noise ratio of node *i* in relation to the overall *SINR* of all nodes inside the cluster. $SINR_{i}$ represents Signal-to-Interference-plus-Noise Ratio of node *i*. *N* illustrates total number of nodes in the cluster.

(4) $$R=\left[\frac{r_{i,j}}{\sum_{i=1}^{N}r_{i,j}}\;\right]\;$$

which gives the distance between two nodes in relation to the aggregate distances across all nodes within the cluster. $r_{i,j}$ represents distance between node *i* and node *j*.

(5) $$H=\left[\frac{HO{P_{count_{i}}}_{,j}}{\sum_{i=1}^{N}HO{P_{count_{i}}}_{,j}}\;\right]$$

which gives the *HoP* count between two nodes in relation to the total *HoP* counts across all nodes within the cluster. $HO{P_{count_{i}}}_{,j}$ illustrates number of hops required to reach from node *i* to node *j*.

(6) $$f_{i}=\sum_{i=1}^{N}\left(\left(W_{1}*E\right)+(W_{2}*\;S\right)+(W_{3}*\left(1-R\right)))+(W_{4}*(1-H))$$

$$W_{1}+W_{2}+W_{3}+W_{4}=1$$

The pseudocode of the Firefly Algorithm is given in Algorithm 1.
**Algorithm 1.** Pseudocode of the Firefly Algorithm
function Objective function $f\left(x\right)$

$u_{i}$ = $i^{th}$ firefly, $i\varepsilon \left[1,n\right]$ ;*n* = number of fireflies;max generation = number of generations of firefly;
$I_{i}$ = light intensity;
Υ = absorption coefficient;Firefly AlgorithmBeginObjective function of $f\left(x\right)$ , $x={\left(x_{1},\ldots \ldots ,x_{d}\right)}^{T}$
Create the starting population of fireflies. $x_{i}=\left(i=1,2,\ldots \ldots ,n\right)$
Light intensity $I_{i}$ at $x_{i}$ is determined by $f\left(x\right)$
Determine absorption coefficient Υ
While (*t* < MaxGen)for *i* = 1:*n*;for *j* = 1:*n*;
$I_{i}$ = Objective Function (fireflies(*i*,:));***if*** $I_{j}$ > $I_{i}$ ;end if Attractiveness changes based on distance *r*;Assess novel solutions and illumination updatebrightness;end for *j*;end for *i*;end whilePost-processing outcomes and visualization;end procedure


The pseudocode SOM method is given in Algorithm 2.
**Algorithm 2.** Pseudocode of SOM method
Initialize:Normalize all input vectors *X*Set SOM grid of size *G* with *M = G_1_ × G_2_* neuronsRandomly initialize weight vector *wⱼ ∈ ℝᵈ* for each neuron *j*, where *d* is dimensionality of *xᵢ*Assign each neuron a grid position *posⱼ ∈ ℝ^2^*for *t* = 1 to *T* doShuffle input vectors *X*for each input vector *xᵢ* doCompute *BMU* (Best Matching Unit):for each neuron *j*, compute distance:
    *distⱼ = ‖xᵢ − wⱼ‖_2_*
11.Find index j* where *distⱼ* is minimized12.Update learning parameters:13.Update weights for each neuron *j*:14.Compute grid distance: d_grid = ‖posⱼ − posⱼ*‖15.Compute neighborhood function:
    Update weight vector:    Assign clusters:
16.for each *xᵢ*, find its *BMU*
17.Assign *xᵢ* to cluster Cⱼ* based on nearest neuron18.end for;19.end for;20.end for;21.end for;


### 3.2. Data Collection

**Figure 2:**
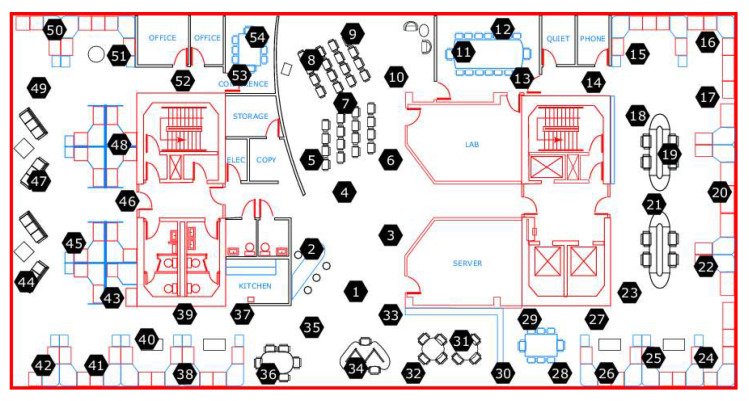
Sensor nodes deployed to collect the Intel Berkeley Research Lab dataset.

The Intel Berkeley Research Lab dataset is an open-source collection compiled at the Intel Berkeley Research Lab. The capturing period spanned from 28 February to 5 April 2004. Figure 2 illustrates that the Intel lab dataset comprises eight features as observational variables, whereas the recorded sensed data instances total 2,313,682. The dataset was gathered utilizing 54 Mica 2 Dot sensors in conjunction with weather sensors deployed at the Intel Berkeley Research Lab. These sensors measure temperature, humidity, and light weather characteristics every 31 s [35].

### 3.3. Data Pre-Processing

**Figure 3:**
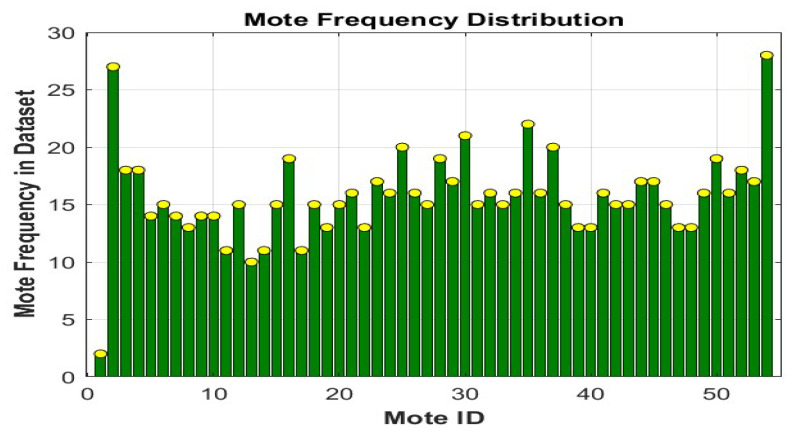
The measurement frequencies of 54 sensors.

To ascertain the precise specifications and total repeats for each sensor, the utilization of each sensor for measuring the temperature, humidity, and light was conducted to facilitate subsequent steps and identify the suitable algorithms and functions that would ultimately enhance the efficacy of the data aggregation strategy, thereby minimizing the aforementioned excess data. Figure 3 illustrates the measurement frequencies of the three occurrences over 54 sensors (Mote IDs).

### 3.4. Distance Calculation

The FOA demands accurate calculation of the distance between two nodes (or fireflies) to ascertain the degree of attraction one firefly exerts upon another (Equation (7)). The appeal of the environment influences the communication of fireflies throughout the search space, guiding them toward the optimal choices.

(7) $$r=\sqrt{{\left(x_{1}-x_{2}\right)}^{2}+{\left(y_{1}-y_{2}\right)}^{2}}$$

where *r* represents the distance between fireflies. Each firefly informally pursues superior solutions by attracting brighter fireflies, as the brightness of a firefly correlates with the effectiveness of the solution it embodies in the optimization problem. The distance calculation ensures that this communication is flexible and responsive to the varying brightnesses of fireflies.

(8) $$d_{ij}=\sqrt{\sum_{k=1}^{d}{\left(P1_{ik}-P2_{jk}\;\right)}^{2}}$$

Employing Equation (8) is essential for calculating the distance between two sets of points, which is fundamental in assessing the relative attractiveness of fireflies. Their advancement within the search space is directed by their allure. As the algorithm progresses, it repeatedly recalculates the distances between fireflies, enabling it to adapt dynamically to their changing positions. This ensures that the inquiry is timely and adept at thoroughly examining the issue space. In this equation, *d* represents the number of dimensions, whereas $P1_{ik}$ and $P2_{jk}$ are the coordinates of fireflies *i* and *j* in the *k*-th dimension [1].

### 3.5. Cluster Formation with Self-Organizing Maps

SOMs are an unsupervised learning approach designed to generate a low-dimensional representation of high-dimensional data while maintaining the data’s topological characteristics. In the realm of IoT sensor networks, SOMs can be utilized to create clusters of sensor nodes according to their attributes, including data patterns, energy levels, and proximities.

Steps for cluster formation with SOMs:

1. Initialization: SOMs commence with a preliminary weight matrix, wherein each sensor node in the SOM is linked to a weight vector that matches the dimensionality of the input data.
2. Input data representation: Each sensor node *i* is denoted by a feature vector:

(9) $$x_{i}=[\;x_{i},\;y_{i},\;E_{res}\left(i\right),\;D_{BS}\left(i\right),\;SINR\;\left(i\right)]$$

$\left(x_{i},\;y_{i}\right)$ represent the node’s position coordinates, $E_{res}\left(i\right)$ is its residual energy, $D_{BS}\left(i\right)$ is the distance to the base station, and $SINR\;\left(i\right)$ is the signal-to-noise ratio.

3. Finding the Best Matching Unit (BMU): For each input vector $x_{i}$ , the SOM determines the BMU, which is defined as the sensor node with the weight vector most analogous to the input vector. Similarity is frequently quantified by the Euclidean distance:

(10) $$distance=∥x_{i}-w_{j}∥=\sqrt{\sum_{k=1}^{d}{\left(x_{ik}-w_{jk}\right)}^{2}}$$

where $x_{i}$ is the input vector for sensor node i ; $w_{j}$ is the weight vector for sensor node j ; d is the dimensionality of the input space; and $x_{ik}$ and $w_{jk}$ are the *k*-th components of the input and weight vectors, respectively.

4. Updating weights: Once the BMU is identified, the weights of the BMU and its adjacent sensor nodes are modified to align more closely with the input vector. The weight update formula is specified as follows:

(11) $$w_{j}\left(t+1\right)=w_{j}\left(t\right)+\alpha \left(t\right).\;h_{j,j^{*}\;}\left(t\right).\;(x_{i}-w_{j}\left(t\right))$$

$\alpha \left(t\right)$ is the learning rate, and $h_{j,j^{*}}\left(t\right)$ is the neighborhood function that determines the influence of the BMU on its neighbors.

5. Iteration: Steps 3 and 4 are reiterated for a predetermined number of iterations or until the weight vectors achieve convergence. Eventually, the sensor nodes will arrange into clusters that reflect the fundamental structure of the input data.
6. Cluster formation: Following training, sensor nodes with analogous weight vectors are combined into clusters. Each cluster signifies a collection of sensor nodes that exhibit analogous properties derived from the attributes utilized in the input vector.

### 3.6. Data Aggregation and Energy Consumption

In conventional IoT sensor networks, duplicate sensor coverage and linked sensing frequently result in redundant data transmission, causing excessive energy consumption and network congestion. Effective data aggregation methods are essential to alleviate these problems. Static clustering or suboptimal CH selection may lead to disproportionate energy usage and diminished network longevity. The proposed method combines SOM-based adaptive clustering with FOA-driven CH selection and data aggregation to solve these issues.

Upon electing CHs, these nodes are tasked with accumulating data from their respective cluster members. The aggregation process aims to eliminate redundant and associated data, thus minimizing the transmission overhead. The CHs subsequently relay the aggregated data to the BS, enhancing the communication energy efficiency and data delivery dependability. The energy for the aggregated data in these nodes is calculated using Equation (12):

(12) $$E_{Da}={lp\;\times E}_{agg}$$

(13) $$E_{agg}=\frac{1}{\sum_{j=1}^{k}{(E}_{CH_{j}})}$$

where lp denotes the packet length, while $E_{agg}$ signifies the energy consumed for aggregated data is calculated using Equation (13). Energy usage is a critically important subject. Effectively managing and depicting the energy consumption of the sensor nodes is essential in the proposed method for accurately estimating the network’s efficiency and longevity. The following formulas are utilized to assess the quantity of energy consumed:

(14) $$E_{TX}\;(lp)={lp\;\times E}_{elec}+\left(lp+E_{agg}\times d^{2}\right)\;if\;d\;<d_{0}$$

(15) $$E_{TX}\;(lp)={lp\times E}_{elec}+\left(lp+E_{tr}\times d^{4}\right)\;if\;d>d_{0}$$

(16) $$E_{RX}={lp\times E}_{elec}$$

where *lp* signifies the duration of the transmitted or received packet, whilst *d* indicates the distance between the destination and the transmitter. The value of $d_{0}$ is ascertained utilizing Equation (17) [1]:

(17) $$d_{0}=\sqrt{\frac{E_{agg}}{E_{tr}}}$$

The energy overhead introduced by cluster formation using the integrated SOM and FOA in IoT sensor networks is estimated to range between 5% and 12% of the total per-round energy expenditure, depending on network density and re-clustering frequency. This overhead arises primarily from data exchanges and network adaptation processes associated with cluster head selection and cluster membership updates. Notably, these additional energy costs are largely offset by improved data aggregation efficiency and reduced redundant transmissions-benefits which translate into extended network lifetime and enhanced overall energy utilization across nodes. Recent simulation-based studies consistently demonstrate that the proposed SOM–FOA hybrid approach achieves superior performance compared to traditional clustering methods, validating the practical value of the incurred overhead in optimizing IoT sensor network operations [1,3,13]. Table 3 summarizes the mathematical notation.

## 4. Simulation and Results

This paper presents a novel hybrid architecture that combines SOMs and the FOA to address the significant challenges of energy efficiency and network longevity in IoT sensor networks. The framework utilizes the adaptive clustering attributes of SOMs and the multi-parameter optimization functions of the FOA to effectively consolidate and transmit sensor data, aiming to minimize redundancy, optimize resource utilization, and enhance practical applicability in modern IoT settings. SOMs are utilized to dynamically cluster sensor nodes based on their spatial placements, similarities in sensed data, and prevailing resource conditions, such as energy levels and connectivity. This produces clusters that are ideally balanced regarding the load and spatial distribution, which is crucial for minimizing redundant transmissions and optimizing network resource efficiency. Subsequent to the establishment of clusters using SOM, the ensuing phase employs the FOA to identify the optimal CHs inside each cluster. The FOA is well-suited for this task due to its ability to discern optimal solutions within complex, multi-parameter contexts.

### 4.1. Simulation Environment

We applied the MATLAB 2024b simulator to conduct data simulation and analysis.

**Figure 4:**
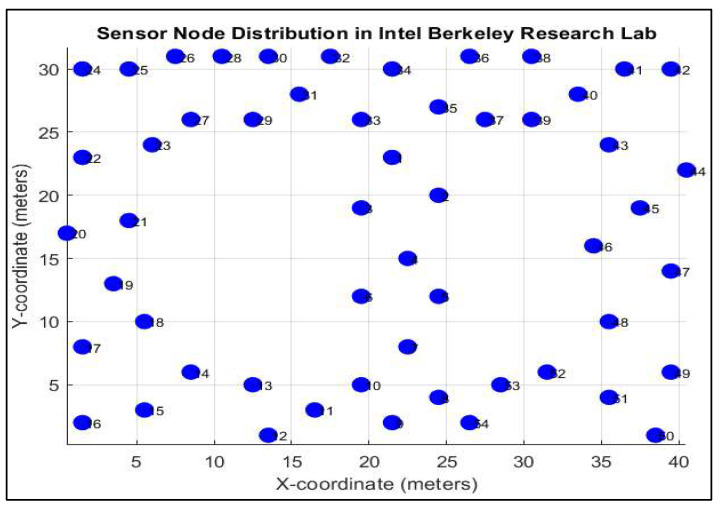
Sensor nodes distribution in Intel Berkeley Research Lab.

Table 4 provides a brief overview of the simulation parameters and variables incorporated in this methodology. Figure 4 depicts the sensor nodes distribution in the Intel Berkeley Research Lab. The value $E_{elec}$ denotes the energy consumed per bit in the transmitter or receiving circuit. The parameters $E_{tr}$ and ${\;E}_{agg}$ are derived from the fundamental model of the sender circuit. The variable $\beta_{0}$ represents the allure of firefly *j* to firefly *i*, which diminishes as the distance between them increases. Typically, $\beta_{0}$ should be a value ranging from 0 to 1. The parameter *γ* dictates the pace at which attraction wanes as the distance from the communicating firefly increases. Generally, it ranges from 0.01 to 100 in most applications. Our proposed FOA uses values of $\beta_{0}$ = 1 and *γ* = 1 for all simulations. a is a parameter utilized for randomization, where a ∈ [0, 1]. In our methodology, the value of a is 0.2. The FOA parameters were selected based on a literature review [1,2,3] and thorough empirical tuning through simulations, crucially balancing exploration and exploitation to optimize convergence speed and solution quality for efficient data aggregation in IoT sensor networks. To assess the effectiveness of the proposed method in comparison with the SOM method, FOA and LEACH, we employ the following metrics: accuracy and error, residual energy, throughput, and network lifetime.

### 4.2. Accuracy and Error Comparison of Algorithms

**Figure 5:**
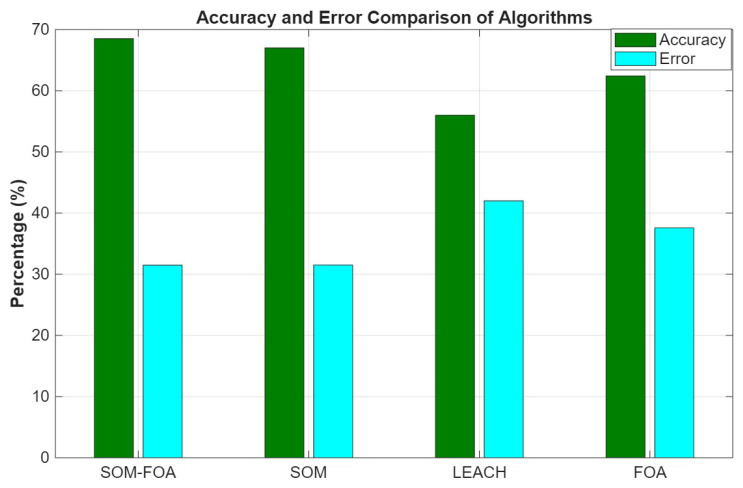
Accuracy and error comparisons of algorithms.

The proposed method, SOM-FOA, demonstrates the highest accuracy of the four, suggesting superior performance in accurately forecasting or aggregating data (Figure 5). The enhanced performance is due to adaptive clustering and optimization approaches that improve data management. Results indicate that the SOM-FOA achieved an accuracy of 68% and an error rate of 31%, while SOM alone showed 67% accuracy and 31% error, demonstrating comparable performance. The minimal difference between SOM-FOA and SOM can be attributed to their shared reliance on SOM-based clustering; however, the integration of the FOA has the potential to deliver greater benefits in more complex or larger-scale network scenarios.

### 4.3. Residual Energy per Round

**Figure 6:**
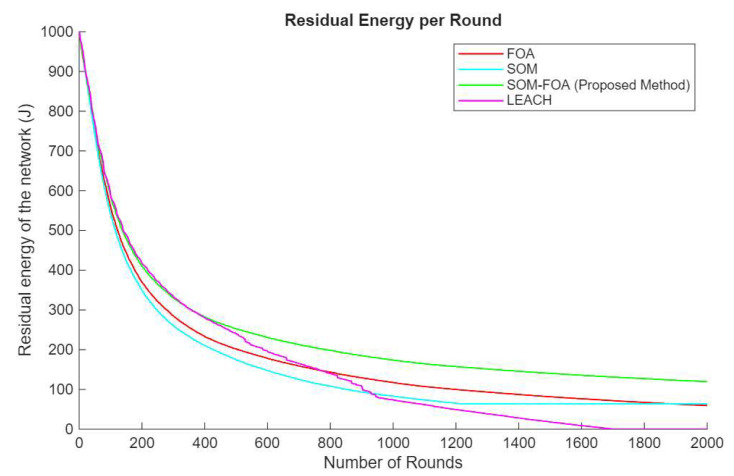
Residual energy per round.

Although SOMs efficiently organize data, energy consumption patterns are contingent upon the network structure and frequency of node activations. Energy is expended in data processing and communication, resulting in a progressive depletion of residual energy as rounds advance. The suggested SOM-FOA method improves the conventional SOM technique by refining the initial clustering procedure. SOM-FOA helps with the selection of the optimal number of clusters and positions for the CHs, leading to more effective energy management. Through the optimization of grouping, SOM-FOA diminishes the energy burden on individual sensors. The SOM-FOA technique has a markedly superior level of residual energy relative to FOA, SOM, and LEACH over the rounds (Figure 6). This signifies that the optimization process using SOM-FOA efficiently distributes the energy load throughout the network.

### 4.4. Throughput with Number of Nodes

**Figure 7:**
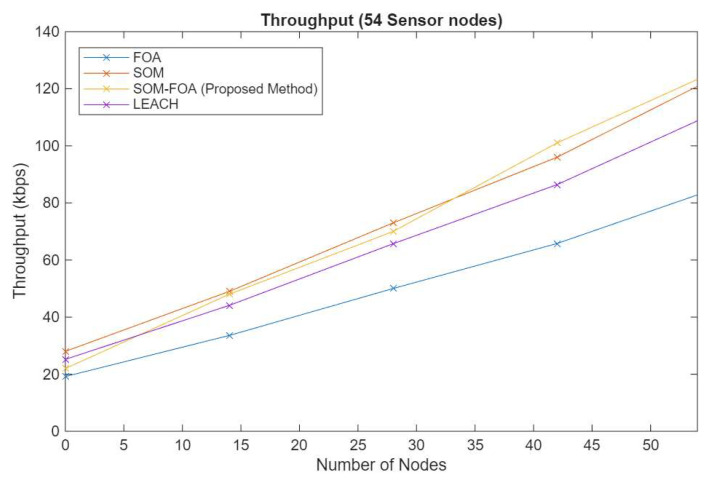
Throughput with number of nodes.

The performance of different algorithms greatly influences how well IoT systems work, affecting factors such as real-time monitoring and energy use. The SOM-FOA approach consistently demonstrates superior throughput across all node quantities. The optimization offered by SOM guarantees the more efficient formation of clusters, resulting in enhanced data transmission rates. Both SOM and SOM-FOA demonstrate exceptional scalability, with the throughput augmented in accordance with the rise in the number of nodes. Nonetheless, SOM-FOA demonstrates enhanced scalability, exhibiting considerable advancements in throughput (Figure 7). The throughput is determined in kilobits per second (Kbps) using Equation (18):

(18) $$Throughput=\;\frac{\left(Number\;of\;bytes\;received\;\times 8\right)}{Simulation\;time}\;\times 1000\;kbps$$

### 4.5. Network Lifetime

**Figure 8:**
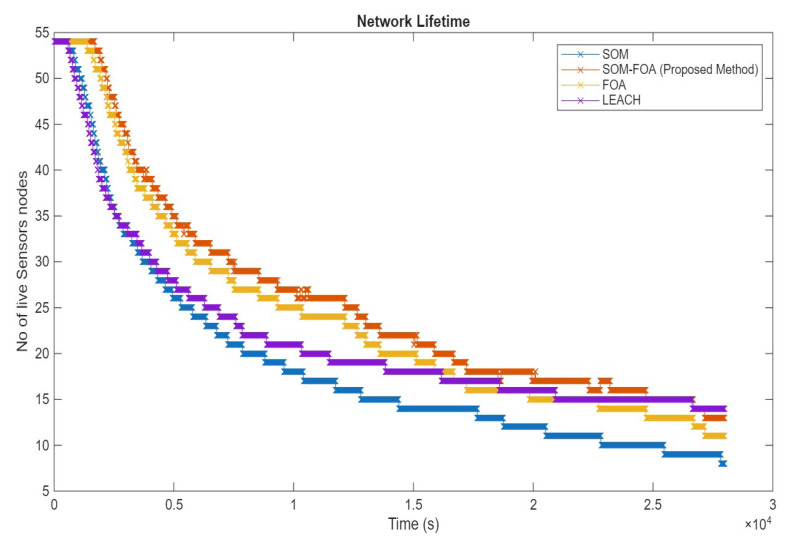
Network lifetime.

The lifetime is a crucial parameter when assessing the performance of IoT sensor networks. It denotes the period in which the network can function efficiently, enabling nodes to interact and execute their sensing activities. The SOM-FOA method integrates SOMs’ advantages with FOA’s optimization potential. This hybrid methodology optimizes the cluster head selection and routing pathways utilizing real-time measurements, hence enhancing the overall network efficiency. The suggested technique consistently adjusts to fluctuating network conditions, facilitating more efficient management of energy resources. This adaptability is crucial for sustaining network performance in dynamic contexts where node circumstances may vary. SOM-FOA exhibits superior performance for network longevity (Figure 8). The network lifetime is ascertained utilizing Equation (19):

(19) $$Network\;Lifetime=\frac{E_{initial}}{E_{total}}$$

(20) $$E_{total}=E_{Tx}\left(l\;p\right)+E_{Rx}\left(l\;p\right)+E_{agg}$$

The principal parameters influencing the network lifetime performance, $E_{initial}$ and $E_{total}$ , denote the initial energy of the nodes and the total energy necessary for data transmission, reception, and aggregation, respectively (Equation (20)).

## 5. Conclusions and Future Work

This research introduces an innovative hybrid framework that enhances data aggregation in IoT sensor networks through the integration of SOMs and the FOA. This integration was motivated by the urgent issues encountered in IoT networks, such as data redundancy, energy efficiency, and the necessity for dependable data transfer. Our research proved that the integration of the adaptive clustering capabilities of SOMs with FOA’s resilient optimization approaches considerably improves the performance of data aggregation.

The experimental research, performed through MATLAB simulations, has yielded persuasive proof of the efficacy of our proposed strategy. The findings indicate a significant decrease in energy usage, enhanced throughput, and prolonged network longevity. The implementation of our approach on the Intel Berkeley Research Lab dataset resulted in a high classification rate, thereby confirming its practical applicability in real-world contexts. Our research highlights the efficacy of hybrid approaches in tackling the complex challenges associated with IoT sensor networks. By utilizing the advantages of both SOMs and the FOA, we have established a foundation for more sophisticated and adaptable data aggregation methodologies. This study paves the way for further research on improving network protocols and increasing the overall effectiveness of IoT systems, helping them reach their full potential in various applications. The applications of the current methodology may be expanded in future to evaluate the efficiency of the proposed approach in comparison with other PSO versions and recent algorithms, or by including statistical measurements. Future research should focus on advancing innovative data aggregation, including IoT scalability and cluster head selection in heterogeneous networks, as well as the applicability of this approach to different contexts or datasets. Concrete future experiments will involve systematic measurement and comparative analysis of the energy consumed during both clustering and data transmission phases, using varied network scales and different re-clustering intervals to robustly validate the energy efficiency and scalability of the proposed SOM-FOA approach.

## Figures and Tables

**Table 1:** Summarization table on the related works.

Reference	Key Technology	Characteristics
[4] 2019	Self-Organizing Map Data Aggregation (SOMDA)	The SOMDA technique excels in handling datasets with high outlier and duplication tendencies, significantly enhancing data reduction and energy efficiency. Its optimized data aggregation approach effectively extends network longevity, outperforming benchmark schemes on all evaluated metrics.
[5] 2022	RBF	The modified RBF algorithm achieves high classification accuracy, reaching 97.54% during training and 97.70% during testing. These enhancements to the hidden layer structure significantly improve data aggregation performance in WSNs, demonstrating the algorithm’s effectiveness and reliability.
[11] 2022	FA-SOM	The FA-SOM algorithm demonstrates competitive cost function values compared to PSO-ACO-K and K-means algorithms on cancer datasets, showcasing its robustness and adaptability. Its superior ranking in the Friedman test further validates its efficiency and effectiveness across diverse datasets.
[12] 2020	Improved Firefly Algorithm (IFA)	The Improved Firefly Algorithm (IFA) significantly extends network lifetime by delaying the death of the first node to 1170 iterations and increases communication efficiency by successfully transmitting 21,000 packets to the base station. It outperforms both the original Firefly Algorithm and LEACH-PSO in prolonging network longevity and reducing power consumption.
[13] 2021	EM-FIREFLY	The EM-FIREFLY method increases network lifetime by approximately 25% and 35% compared to Algorithm-PSO and optimal clustering methods, respectively. It also efficiently manages power consumption, outperforming other approaches in extending the operational duration of WSNs.
[14] 2022	Hybrid Aquila Optimizer and Firefly Algorithm (HAOFA)	The HAOFA routing model improves energy efficiency in IoT networks by intelligently selecting and evenly distributing cluster head nodes. This strategy significantly reduces power consumption and extends the overall network lifetime.
[15] 2021	SOM	The SOM algorithm significantly reduces the number of transmitted packets in WSNs, effectively lowering traffic load and conserving sensor energy. This data aggregation approach improves overall network performance by enhancing energy efficiency and increasing the accuracy of the collected data.
[16] 2021	FAJIT	The FAJIT algorithm leverages fuzzy logic-based parent node selection and min–max normalization to enhance WSN performance. It achieves about 40% better average aggregation factor than the Distributed Algorithm for Integrated tree Construction and data Aggregation (DICA), with shorter schedule lengths and lower energy consumption during data transmission.
[17] 2022	ACNM	The ACNM algorithm stands out due to its superior performance in reducing the packet dropping ratio, routing overhead, network delay, and testing errors while improving throughput and accuracy. It minimizes computation periods and adapts transmission rates based on node capabilities, resulting in lower latency and more reliable data transmission compared to ELM.
[18] 2020	RSC	The RSC algorithm outperforms fan-shaped clusters by offering better scalability and longer network lifetime, especially in large-scale sensor deployments. It achieves this through improved load balancing and energy efficiency by dividing the deployment area into virtual concentric rings and sectors, applying angular routing to minimize hops and reduce energy consumption.
[19] 2022	K-means	The extended weighted K-means approach is highly effective for healthcare data analysis, as it assigns weights to data points to assess patient criticality and prioritize information while avoiding redundancy. Its performance is validated through key performance indicators (KPIs), demonstrating improved clustering feasibility and accuracy for healthcare applications.
[20] 2020	Hierarchical Clustering	The use of multiple sinks in WSNs reduces energy consumption, increases throughput, and significantly improves the packet delivery ratio (PDR). This approach outperforms traditional single-sink systems by enhancing overall network efficiency, reliability, and scalability through better load distribution and reduced communication bottlenecks.
[21] 2019	Energy Efficient Data Aggregation Scheme (EEDAC)	The EEDAC-WSN algorithm improves WSN stability by 17.67%, with the EEDAC-WSN-Silent variant achieving a 23% increase. This improvement is driven by minimizing intra-cluster communication using metadata frames and enabling direct communication to the base station for nodes close to it, resulting in extended network lifespan and enhanced throughput.
[22] 2019	LDT	The LDT scheme offers a linear time complexity of O(n), performing better than LEACH and EACCC in overall results. Its multipath clustering strategy delivers high scalability, low delay, reduced overhead, low energy consumption, and minimized traffic load, making it well-suited for efficient WSN operations.
[23] 2021	Hierarchical Routing	This method improves packet delivery rate significantly compared to rendezvous-based routing and binary tree-based aggregation. It also reduces energy consumption by 20% relative to rendezvous-based routing and by 28% compared to binary tree-based aggregation, resulting in more efficient and reliable WSN operation.
[24] 2019	PUDCRP	The PUDCRP protocol significantly enhances network lifetime by delaying the first node death and increasing the time at which half the nodes expire. It maintains balanced energy consumption with higher average residual energy and reduces energy consumption per round, leading to overall improved network performance and efficiency.
[25] 2019	EC-PSO	The EC-PSO algorithm enhances energy efficiency and network lifetime by effectively avoiding energy holes and balancing energy consumption through a specialized clustering and protection approach. It outperforms VD-PSO by extending the network lifetime to about 900 rounds before the first node dies, significantly improving overall network performance.

**Table 2:** Comparison of data aggregation techniques [33,34].

Techniques	Strategy	Redundancy	Accuracy	Avg. Energy Consumption
DBST	Tree Based	Moderate	Moderate	Less
SDRE	Tree Based	Less	High	Less
BHCDA	Cluster Based	Moderate	Less	Less
REDD	Cluster Based	Less	Moderate	Less
EERDAT	Cluster Based	Less	High	Less
EEBCDA	Cluster Based	Less	High	Less
DEAD	Tree Based	Less	Moderate	Moderate
LEACH	Cluster Based	Moderate	Moderate	Less
SOM-FOA Proposed Method	Cluster Based	Less	High	Less

**Table 3:** Summary of mathematical notation.

Υ	Absorption coefficient
*I*	Light intensity
β	Attractiveness
r	Distance between fireflies
a	Randomization parameter in the range a ∈ [0, 1]
rand	Stochastic variable evenly distributed throughout the range [0, 1]
$E_{agg}$	Energy used for aggregated data
$E_{elec}$	Consumed energy for each bit
$E_{tr}$	Energy used for transmission

**Table 4:** Simulation parameters.

Parameter	Value
Number of nodes	54
Number of rounds	2000
Initial node energy	2 J
$E_{elec}$	5 nj/bit
$E_{agg}$	10 pJ/bit/m^2^
$E_{tr}$	0.0013 pJ/bit/m^4^
$\alpha_{0}$	0.2
$\beta_{0}$	1

## Data Availability

The original contributions presented in this study are included in the article. Further inquiries can be directed to the corresponding author.
